# Impact of Complete Denture and Mandibular Advancement Device in the Management of Completely Edentulous Obstructive Sleep Apneic Individuals: A Systematic Review with Meta-Analysis

**DOI:** 10.30476/dentjods.2022.93891.1743

**Published:** 2023-03

**Authors:** Preetha Krishnamurthy, Fathima Banu, V Anand Kumar

**Affiliations:** 1 Past-Postgraduate, Dept. of Prosthodontics and Crown & Bridge, Sri Ramchandra Institute of Higher Education & Research, Sriher, India; 2 Dept. of Prosthodontics and Crown & Bridge, Sri Ramchandra Institute of Higher Education & Research, Sriher, India.

**Keywords:** Airway obstruction, Edentulous mouth, Complete denture, Mandibular Advancement, Sleep Apnea, Obstructive

## Abstract

**Statement of the Problem:**

Obstructive sleep apnea (OSA) is an underdiagnosed and potentially serious disorder that is accentuated by edentulism. The overclosure of the mandible and a potential upper airway collapse during sleep creates challenges in treating edentulous sleep apneic patients.

**Purpose:**

To evaluate complete dentures and mandibular advancement devices as potential oral appliances in the management of sleep apnea in completely edentulous patients.

**Materials and Method:**

The study design was a systematic review with meta-analysis. The search criteria complied with the preferred reporting items for systematic reviews and meta-analyses (PRISMA) guidelines and the keywords in population, intervention, control, and outcomes (PICO) format was systematically searched for relevant research articles published till August 2021 in an electronic database (PubMed, Cochrane, Science Direct, Ovid). Randomized controlled trials and cohort studies were included that compared the effectiveness of oral appliances on apnea-hypopnea index (AHI), airway space, and quality of sleep in edentulous sleep apneic patients.

**Results:**

1785 articles were derived from the initial search and based on inclusion criteria, 10 articles were systematically filtered for qualitative analysis and assessed for risk of bias using the Cochrane risk of bias tool and ROBINS-I tool. Out of the 10 articles, 5 articles were taken for quantitative analysis. The use of a mandibular advancement device (MAD) showed a decrease in AHI score, but the available data was heterogeneous to conduct a meta-analysis. The mean difference of AHI for the random effect model between the non-complete denture and complete denture wearers at sleep was -0.49[95% CI (-1.47,0.48)] events per hour, but the change was non-significant (*p*>.05).

**Conclusion:**

The complete dentures as an oral appliance had reduced apneic episodes in completely edentulous sleep apneic patients, but the effectiveness cannot be solely attributed to the prosthesis in the treatment of OSA. MAD showed greater improvement in reducing AHI, however, the level of evidence was inadequate to provide a conclusive statement.

## Introduction

Aging is accompanied by changes in the quality, quantity, and pattern of sleep [ 1 ]. It has been estimated that about 50% of elderly adults experience sleep disturbance, and epidemiologic evidence suggests that this impaired the ability to initiate, maintain, and ultimately achieve optimal sleep [ 2 - 4 ]. Sleep impairment could be a marker of increased mortality and neurocognitive dysfunction [ 2 - 4 ]. Disturbed sleep is commonly associated with disordered breathing that ranges from intermittent, partial obstruction of the airway like snoring to repeated obstruction of the upper airway such as obstructive sleep apnea (OSA) syndrome [ 5 ]. The obstructive sleep apnea syndrome or obstructive sleep apnea/hypopnea syndrome is characterized by episodes of apnea and hypopnea during sleep, snoring, and daytime sleepiness [ 6 - 9 ].

Untreated OSA leads to excessive daytime sleepiness, tiredness, impairment of concentration, cardiovascular diseases such as myocardial infarction, stroke, diabetes mellitus, impaired cognitive functions, and depression [ 10 ]. Additionally, OSA patients are more likely to be involved in motor vehicle crashes and have an increased risk of mortality compared to individuals without OSA [ 10 - 16 ]. Early recognition and treatment of OSA may prevent adverse health consequences. Thus, treatment of OSA requires extensive communication and strong collaboration between the physician and patient to develop the most effective individual treatment plan. Approximately 28–67% and 20–54% of completely edentulous men and women respectively were known to suffer from OSA that would exacerbate their apneic condition [ 9 ].

Complete edentulism changes the anatomy of the upper airway during sleep by the influence of the postural rest position of the mandible, the tongue, and the muscle tone [ 1 , 17 ]. The decreased occlusal vertical dimension following the loss of teeth leads to a reduction in the lower facial height, and rotation of the mandible with a reduction in the airway space [ 17 - 19 ]. These physiological changes along with progressive alveolar bone resorption led to overclosure of the mandible and a potential upper airway collapse. A decrease in posterior air-way space and/or the hypotonicity of the pharyngeal musculature in edentulous individuals were also found to increase airway resistance and aggravate OSA [ 5 , 19 - 20 ]. Impending speculation continues to exist regarding the association between edentulism and sleep apnea [ 20 - 21 ].

The treatment modalities for OSA include surgical and nonsurgical methods, however, evidence regarding surgical management and their outcomes are limited in edentate individuals [ 22 ]. Nonsurgical protocols include behavioral management, continuous positive airway pressure therapy, and oral appliances [ 6 ]. Continuous positive airway pressure reduced mortality in older patients and leads to a long-term good prognosis [ 23 ]. However, adherence to the therapy in the elderly was impaired by factors such as cognitive impairment, medical and mood disturbances, nocturia, lack of a supportive partner, and impaired manual dexterity [ 24 ]. In addition, the edentulous patients were found to have difficulties in using continuous positive airway pressure due to loss of retention and lack of compliance in absence of teeth. On the contrary, the use of oral appliances such as a mandibular advancement device maintained the patency of the upper airway and prevented the lumen of the pharynx from collapsing in mild-to-moderate dentate cases [ 25 - 26 ]. The complete denture that reestablishes the lost vertical dimension and the muscle engram can also be a potential oral appliance in opening the airway space [ 2 ]. However, the clinical practice of utilizing complete dentures as a treatment appliance in OSA patients is still questionable. Moreover, edentulous patients may not be ideally suited for treatment with oral appliances owing to inadequate intraoral retention, dry mouth, increased salivation, tooth soreness, and jaw muscle or jaw joint discomfort that led to discontinuation of the appliance [ 26 - 27 ]. Hence, a systematic review was conducted to identify the highest level of evidence from the available literature to utilize complete dentures in the treatment of OSA. The objective of the review is to analyze the prognostic effectiveness of complete dentures and mandibular advancement devices in the management of apnea and improvement of airway space and quality of sleep for an edentulous sleep apneic individual.

## Materials and Method

### Protocol

The protocol was designed following the Cochrane standards for systematic reviews. The search criteria complied with the preferred reporting items for systematic reviews and meta-analysis protocols (PRISMA) guidelines. The literature search was done using the PICOS strategy including population( completely edentulous sleep apneic patients), intervention [complete denture, implant-supported complete denture, mandibular advancement device (MAD)], comparison (edentulous state), primary outcome [apnea-hypopnea index (AHI)], secondary outcome (airway space, quality of sleep), and study design( randomized controlled trials, cohort studies).

### Criteria for the inclusion of studies

The inclusion criteria were randomized controlled trials and cohort studies that investigated the effect of removable dentures, implant-supported dentures, and MAD on the airway space, AHI, and the quality of sleep in the completely edentulous sleep apneic individuals. The study design that was able to answer the research question with a lower risk of systematic errors and greater control over potential confounding variables was included. The preliminary scoping review revealed the predominance of cohort study design that constituted the fourth-best scientific evidence to support the effectiveness of therapeutic interventions and their prognosis. Hence, the systematic review included both randomized controlled trial and cohort study design for the search strategy. Review articles were excluded from the study.

### Search strategy

The database till August 2021 was searched including PubMed, Science Direct, Ovid, Cochrane database of systematic reviews. Articles were hand-searched from specialty journals such as Journal of Prosthetic Dentistry, Journal of Prosthodontic Research, Journal of Advanced Prosthodontics, and from the reference of relevant articles obtained at the end of the database search. The electronic search included all the possible randomized controlled trials and cohort studies. The reference lists of the research articles were also checked for possible inclusion of data. The search terms (Medical Subject Heading) were edentulous, edentulism, edentate, edentulousness, edentulous patient, edentulous population, missing teeth, absence of teeth, complete edentulous, sleep apnea, obstructive sleep apnea, OSA, obstructive sleep apnea syndrome, sleep-disordered breathing, denture, complete denture, removable complete denture, maxillary complete denture, mandibular complete denture, mandibular advancement device, MAD, modified mandibular advancement device, airway, airway space, airway volume, AHI, apnea-hypopnea index. The Boolean words OR, AND were used between the terms to obtain relevant articles that were sorted by pubsolr12.

### Study eligibility and data extraction

Two authors evaluated all retrieved articles and the third author resolved the disagreements. Irrelevant records (abstracts not available, bibliographic reviews, descriptive studies, animal studies) were excluded, and the full texts of potentially relevant studies were examined to answer the question of interest. The relevant articles that met the inclusion criteria were analyzed and the data was extracted in Table 1 based on (1) author and year of study, (2) demographic characteristics (age, sample size), (3) method of assessment, (4) time of assessment, (5) type of intervention, (6) outcome variable (airway space /AHI /quality of sleep), and (7) overall interpretation.

**Table 1:** Basic characteristics of the included studies

S.No	Author and year, type of study	Demographic details	No of participants	Time of assessment	Methods used	Outcome variables	Intervention	Overall interpretation
1.	Gowda M *et al*. [ 1 ] 2016 COHORT	50-70 years (average 63 yrs.)	30 completely edentulous patients with symptoms of snoring	6 weeks to 8 weeks after wearing of dentures	Cephalometry	Airway space	Complete denture	Wear of the complete dentures during the night did not improve the airway space.
					Overnight pulse oximetry	Oxygen saturation		
					ESS	Excessive daytime sleepiness		
2.	Milosevic B *et al*. [ 28 ] 2016 Cohort	50-65 years	9 edentulous patients with OSA	3 months after prosthetic treatment	ESS	Quality of sleep	Complete denture	Rehabilitation of edentulous patients with complete dentures that has an optimal vertical dimension of occlusion increased the diameter of the upper respiratory tract, which was reflected in a significant reduction of OSA syndrome symptoms and AHI score.
					PSG	AHI		
					Magnetic resonance imaging (MRI)	Airway diameter		
3.	Bucca C *et al*. [ 24 ] 2006 COHORT	Mean age- 69 ± 9 years	48 edentulous subjects	No available data	PSG	AHI	Complete denture	Retropharyngeal space was significantly decreased by the removal of the complete dentures. The author states that the advantages of denture removal during sleep should be weighed against the risk of favoring upper airway collapse.
					Cephalometry	Upper airway size		
					Spirometer	Forced mid-inspiratory airflow rate.		
					Chemiluminescence analyzer	Exhaled nitric oxide and oral nitric oxide		
4	Tripathi A *et al*. [ 22 ] 2016 Cohort	Mean age- 61 ± 4 years	17 edentulous patients who had worn complete dentures for at least 1 year	6 months after wear of MAD	ESS and Berlin questionnaire.	Excessive daytime sleepiness	Complete denture modified to MAD	An increase in the pharyngeal volume affected by complete dentures was pronounced with modification into a MAD. The increases in volume in the velopharynx region, followed by hypopharynx and oropharynx were observed with a significant decrease in the AHI after 6 months of use of MAD during sleep at night.
					y PSG	AHI		
					Cone-beam computed tomography	Airway volume		
5	Chen Q *et al*. [ 30 ] 2017 Cohort	Mean age- 67.4 years	30 edentulous patients wearing dentures for at least 6 months	7 days after the new denture wear	PSG	AHI, lowest oxygen saturation (L-SpO2),	Complete denture	The average AHI for all 30 participants was significantly higher when they slept with dentures than without dentures. The authors concluded that the wear of dentures can lead to a significant increase of AHI among edentulous people.
					Pittsburgh Sleep Quality Index (PSQI)	Sleep quality and disturbances		
					ESS	Excessive daytime sleepiness		
6	Endeshaw YW *et al*. [ 26 ] 2004 Cohort	Age> 64 years	58 edentulous subjects	No data available	Overnight ambulatory sleep recording	AHI, Sleep-disordered breathing	Complete denture	A significant association between sleep-disordered breathing and denture use was evident. Subjects were found to have worsening of AHI and a decrease in their anteroposterior oropharyngeal wall distance when examined without their dentures. However, there was insufficient statistical data to detect any significant difference in this regard.
7	Tripathi A *et al*. [ 33 ] 2019 Cohort	60-65 years of age	183 edentulous patients	3,6,9 months intervals	BERLIN questionnaire and ESS	Sleep disordered breathing	A complete denture, modified to MAD	Use of complete dentures modified to function as MAD led to a reduction in AHI. An inverse correlation between OSA severity and serum serotonin level was also observed.
					PSG	AHI, Respiratory effort-related arousals, and Respiratory disturbance index		
					Cephalometry, and intraoral examination	The skeletal and soft tissue profile record		
					Serum serotonin	Biomarker for respiratory function and sleep-disordered breathing		
8	Hoekema A *et al*. [ 32 ] 2007 Cohort	Mean age- 55.6 years	6 edentulous OSA patients.	6 months to 24 months	PSG	AHI	2 implant-retained mandibular overdentures converted to mandibular repositioning appliance	2 implant-retained mandibular overdentures converted to is a viable treatment modality in edentulous OSA patients in reducing AHI.
9	Almeida FR *et al*. [ 29 ] 2012 Cohort	Mean age-69.6 years	23 edentulous patients	15 days	Previous dentures questionnaire (PSQI)	Sleep quality and disturbances	Complete denture	OSA patients may experience more apneic events if they sleep with their dentures in the oral cavity.
					ESS	Excessive daytime sleepiness		
					PSG	AHI		
10	Emami *et al*. [ 31 ] 2021 RCT	65 years or older	70 edentulous patients	1 month	PSG	AHI, SpO_2_	Complete Denture	Edentulous elders have good sleep quality, independent of nocturnal prosthesis wear. The removal of dentures during night-time were recommended
					ESS	Excessive daytime sleepiness		
					Pittsburgh Sleep Quality Index questionnaire	Perceived sleep quality		
					Oral health impact profile-20	Oral-health-related quality of life		

### Statistical Analysis

Cochrane Rob-2 tool, and ROBINS-1 tool was used for the risk of bias assessment. The meta-analysis was done using a random effect model to calculate the pooled incidence rates and their 95% confidence intervals (CI). Heterogeneity between the studies was evaluated by I^2^ statistics.

## Results

### Search results

**Figure 1:**
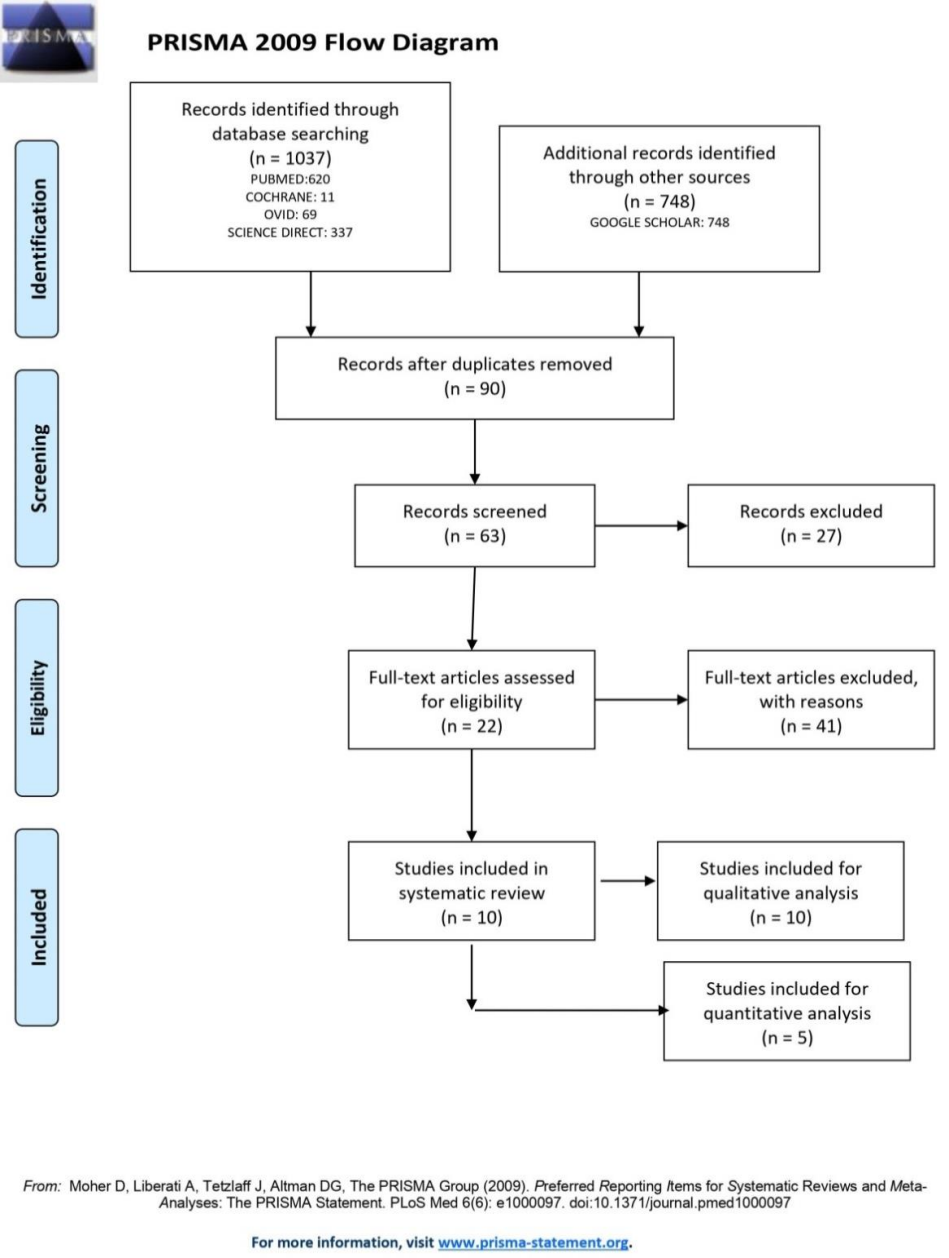
Preferred Reporting Items for Systematic Reviews and Meta-Analyses (PRISMA) Flowchart

A total of 1785 articles were retrieved using databases from online sources. After the elimination of duplicates (n=1695), 63 titles and abstracts were reviewed, of which 41 were excluded that were not based on inclusion criteria (designs without intervention, reviews, or dentate patients). A total of 22 articles were evaluated in detail to determine their eligibility, and 10 met the criteria for inclusion in the systematic review. The flow chart following the PRISMA criteria and model of the complete search and inclusion process of the studies is illustrated in (Figure 1). 

### Risk of bias in the included studies

To determine the validity of the included studies, the Cochrane risk of bias tool for randomized controlled trials (Table 2) and a ROBINS-I tool were used to assess the risk of bias in the selected cohort studies (Table 3) using particular parameters including confounding bias, selection bias, measuring interventions, a departure from interventions, missing data, measuring outcomes, reporting bias, and overall bias. Among the 4 articles that assessed the airway space, 2 articles reported a low risk of bias, and among the 9 articles that assessed AHI, 5 articles reported a low risk of bias. 

**Table 2:** Risk of Bias - ROBINS-I Tool

Study	Confounding Bias	Selection Bias	Measuring Interventions	Departures from interventions	Missing data	Measuring outcomes	Reporting bias	Overall bias
Gowda M *et al*. [ 1 ] 2016	Low	Low	Low	Low	Low	Low	Low	Low
Milosevic B *et al*. [ 28 ] 2016	Moderate	Moderate	Low	Low	Low	Moderate	Low	Moderate
Bucca C *et al*. [ 24 ] 2006	Low	Low	Low	Low	Low	Low	Low	Low
Tripathi A *et al*. [ 22 ] 2016	Low	Moderate	Low	Low	Low	Low	Low	Moderate
Chen Q *et al*. [ 30 ] 2017	Low	Low	Low	Low	Low	Low	Low	Low
Endeshaw YW *et al*. [ 26 ] 2004	Low	Low	Low	Low	Moderate	Serious	Low	Serious
Hoekema A *et al*. [ 32 ] 2007	Low	Moderate	Low	Moderate	Moderate	Low	Moderate	Serious
Almeida FR *et al*. [ 29 ] 2012	Low	Low	Low	Moderate	Moderate	Low	Low	Moderate
Tripathi A *et al*. [ 33 ] 2019	Low	Low	Low	Low	Low	Low	Low	Low

**Table 3:** Risk of Bias- Cochrane Risk of Bias Tool

Study	Random Sequence Generation	Allocation Concealment	Selective Reporting	Other Bias	Blinding of Participants and Personnel	Blinding of Outcome Assessment	Incomplete Outcome Data
Emami *et al*. [ 31 ] 2021	Low	Low	Low	Low	Low	Low	Low

### Description of the studies

The characteristics of the included studies (n=10) are presented in Table 1. The airway space was assessed in 4 of the 10 research articles, AHI was assessed in 9 of the 10 research articles, and quality of sleep was as assessed in 5 of the 10 research articles. The cumulative data is represented in Table 4. Based on the descriptive data synthesis, 5 articles were included only for qualitative analysis, and 5 articles for both qualitative and quantitative analysis. The mean age group of the patients included ranged from 55 to 77 years.

**Table 4:** Apnea-Hypopnea Index (AHI) score and the airway space volume before and after wear of the prosthesis (* indicates statistical significance)

Author	Type of Intervention	AHI		Airway space/volume	
		Without denture	With denture	Before denture	After denture
Endeshaw *et al* [ 26 ] 2004	Complete Denture	5-14	≥15 *	Not assessed	
Bucca *et al* [ 24 ] 2006	Complete Denture	17.4±3.6*	11.0 ± 2.3*	Retropharyngeal space -12.7±4.2 mm *	15.2±3.3mm *
				Posterior Airway Space 4.8 ± 0.8 mm	6.9±1.0mm
Hoekema *et al* [ 32 ] 2007	Mandibular repositioning appliance (each patient individually assessed)	5.8 to 18.3	0 to 12.8	Not assessed	
Almeida *et al* [ 29 ] (2012)	Complete Denture	19.9±10.2*	25.9 ± 14.8 *	Not assessed	
Gowda *et al* [ 1 ] (2016)	Complete Denture	Not assessed		Superior Airway Space=8.96±1.84mm	8.90±1.79mm
				Middle Airway Space=7.00±1.59mm *	7.30±1.46mm *
				Posterior Airway Space 11.76±1.83mm	11.93±2.08mm
Milsoveic *et al* [ 28 ] (2016)	Complete Denture	14.84± 7.54*	7.43 ± 4.92*	Oropharynx = 9.31±2.44mm *	11.97±2.74mm*
				Velopharynx =5.18 ± 1.62* mm *	8.54±2.25mm*
				Uvula sagittal = 3.88 ± 2.66 mm *	8.22±1.80mm *
				Uvula axial (anteroposterior)=4.92±2.46mm*	8.49±2.91mm *
				Uvula axial (latero-lateral)=16.46±4.49mm*	19.48±3.74mm*
Tripathi *et al* [ 22 ] (2016)	MAD	21.76±4.41*	5.41± 2.50*	4.19 cm^3^ (With denture) *	6.58cm^3^ (Denture to MAD) *
Chen *et al* [ 30 ] (2017)	Complete Denture	13.4±14.0*	16.3 ± 14.7*	Not assessed	
Tripathi *et al* [ 33 ] (2019)	MAD	Score not mentioned	Score not mentioned*	Not assessed	
Emami *et al* [ 31 ] 2021	Complete Denture	25.6 (16.4)	26.6 (17.9)	Not assessed	

### Primary Outcome Measures-AHI

A total of 447 patients were evaluated across 9 articles, and polysomnography (PSG) was the common standard tool that assessed AHI. The complete denture was the intervention in 6 of the 9 research articles. Buccal *et al*. [ 24 ] and Milsoveic *et al*. [ 28 ] observed a significant reduction in AHI with complete dentures (11.0±2.3 & 7.43±4.92 respectively) that was compared without dentures (17.4±3.6 & 14.84±7.54 respectively). On the contrary, the presence of complete dentures had increased the AHI in 3 of the 9 research articles evaluated. Almeida *et al*. [ 29 ], Chen *et al*. [ 30 ], and Emami *et al*. [ 31 ] observed increased AHI (25.9±14.8, 16.3±14.7, and 26.6 ±17.9 respectively) with complete dentures compared to (19.9±10.2, 13.4±14.0 and 25.6±16.4 respectively) without dentures. Endeshaw *et al*. [ 26 ] found no statistically significant differences in the AHI that was assessed during ambulatory sleep between the partial and full dentures wearers, either upper or lower denture wearers, and both upper and lower denture wearers. 

MAD was the intervention in 3 of the 9 research articles that assessed AHI. Hoekema *et al*. [ 32 ] identified that PSG conducted in ambulatory edentulous OSA individuals rehabilitated with mandibular implant overdentures modified into mandibular repositioning appliance (MRA) resulted in the reduction of AHI (AHI< 5). Tripathi *et al*. [ 22 ] (2016) noticed a significant decrease in the AHI from 21.76 to 5.41 after 6 months of wear of a complete denture modified into a MAD during sleep. In 2019, Tripathi *et al*. [ 33 ] observed that the improvement in AHI scores was more appreciable between the 6th- to 9th-month intervals compared to the 3-months outcome with the complete denture modified into MAD.

### Estimating the impact of the complete denture on the apneic index

**Figure 2:**
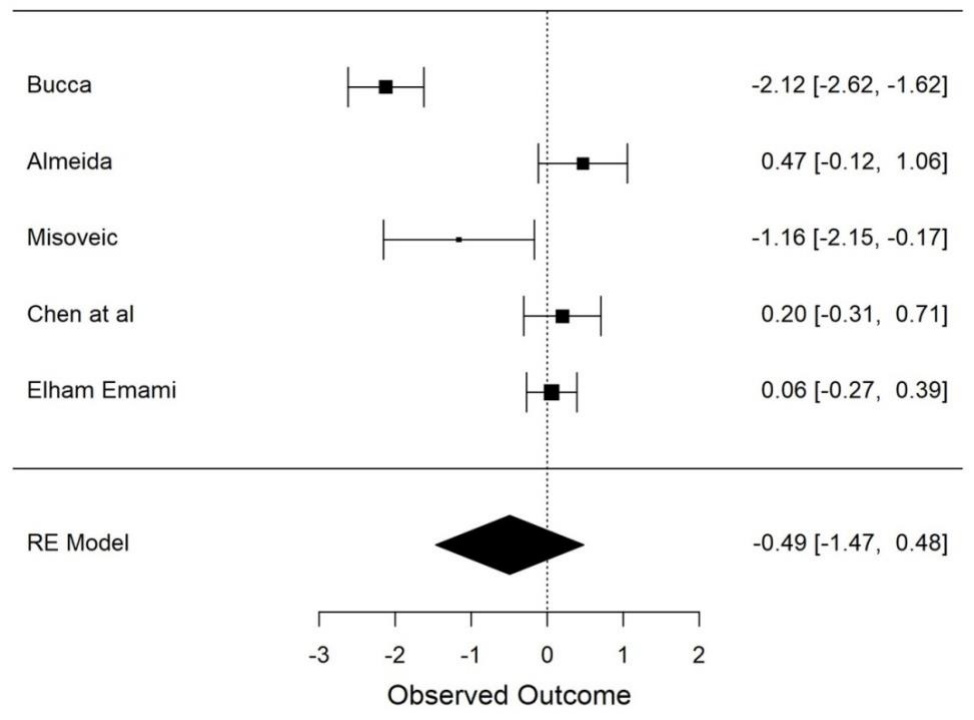
Influence of complete denture in apnea-hypopnea index (AHI)

A total of 5 studies that evaluated the AHI index using PSG before and after denture wear were taken for quantitative meta-analysis [ 24 , 28 - 31 ]. The mean difference for the random effect model between the non-denture wearers and denture wearers was -0.49[95% CI (-1.47, 0.48)] with the diamond tracing towards the treatment effect (Figure 2). The meta-analysis revealed that denture wear reduced AHI, but the change was not significant (*p*< .05). There was a higher risk of heterogenicity (I^2^=94.252%) and the certainty of the evidence was considered low.

### Secondary Outcome: Airway Space

104 patients in the 4 selected articles were evaluated for the airway space. The airway space assessment in 2 of the 4 research articles used cephalometric analysis. Bucca *et al*. [ 24 ] (2006) observed the retropharyngeal space had significantly decreased (1.27±0.42cm) without complete denture wear when compared to denture wear (1.522±0.33cm) during sleep, indicating that the absence of denture increased the upper airway obstruction. However, Gowda *et al*. [ 1 ] (2016) observed no significant improvement in the airway space with dentures (superior airway space 8.90mm±1.79, middle air way space 7.30mm±1.46, posterior airway space 11.9 mm±2.08) and without dentures (superior airway space 8.96mm±1.84, middle airway space 7.00mm±1.59, posterior airway space 11.76mm±1.83). In consensus with Bucca *et al*. [ 24 ], Milsoveic *et al*. [ 28 ] in 2016 observed a reduction in the symptoms of OSA due to a significant increase in the airway diameters of the oropharynx, velopharynx, and uvula regions of the patients wearing complete dentures using sagittal and axial tomograms. Tripathi *et al*. [ 22 ] (2016) used cone-beam computed tomography (CBCT) to evaluate the airway volume in edentulous patients wearing dentures that was modified into MADs and observed a statistically significant increase in the airway volume when compared with unaltered complete dentures, or without any prosthesis.Secondary Outcome: Quality of Sleep

Quality of sleep was evaluated using Epworth sleep scale (ESS). Gowda *et al*. [ 1 ] found that there was no significant difference in ESS with (8.83±1.81) and without complete denture (8.83±1.64). Similarly, Emami *et al*. [ 31 ] also found that the mean paired difference observed between ESS scores with (6.1±4.1) and without dentures (6.3±4.3) at sleep was marginal without significant improvement. Milsoveic *et al*. [ 28 ] qualitative measurements reported that the quality of sleep improved from good to excellent in majority of the participants. Chen *et al*. [ 30 ] stated that only 23.3% of the study population had an ESS score of more than 10 after wearing dentures. In contrast with the above results, Almeida *et al*. [ 29 ] noted that the daytime dysfunction with ESS increased by more than 10 in 21.7% of patients before denture wear, whereas it increased in 50% of the patients after denture wear. In the studies conducted by Tripathi *et al*. [ 22 - 33 ] with MAD, the ESS questionnaire was used only as a diagnostic aid.

## Discussion

A consistent number of elderly people are at a risk of OSA aggravated by edentulism and, consequently related to morbidity and mortality [ 9 ]. Continuous positive airway pressure has been the best method of management, but a lack of compliance has reduced its usage and made health care specialists lookout for other methods of management [ 23 ]. In edentulous patients, complete dentures and oral appliances like mandibular advancement devices, tongue retaining, and positioning devices are intended to restore natural anatomy thus benefitting sleep apneic patients. However, their effectiveness is unclear. Wear of dentures during sleep was proposed to increase the retroglossal space and prevent or reduce OSA in edentulous patients [ 4 ]. This systematic review was aimed to evaluate the effect of complete dentures and MAD on the quality of sleep, airway space, and AHI in edentulous sleep apneic patients. The literature search contained numerous case reports on complete dentures and MAD in treating edentulous apneic patients. However, they were not included in this systematic review, as they constitute a low level of scientific evidence. 

The review revealed that the removal of complete dentures at night decreased the retropharyngeal space significantly, and the advantages of removing dentures during sleep should be weighed against the risk of favoring upper airway collapse in an apneic individual [ 24 , 28 ]. A contradictory outcome was also observed in completely edentulous sleep apneic subjects with no significant differences in the airway on wearing complete dentures during sleep [ 1 ]. The differed results from the review could be due to the varied selection criteria and the methodology, wherein Bucca *et al*., [ 24 ] included the patients based on AHI score taken with PSG while Gowda *et al*. [ 1 ] included the OSA patients based on their chief complaint of snoring. Moreover, the conflict in results could be due to the method of assessing the patients; Bucca *et al*. [ 24 ] evaluated lateral cephalograms taken in a supine position while Gowda *et al*. [ 1 ] evaluated them in a natural head position. Owing to such differences and inadequate evidence, there is uncertainty regarding whether complete dentures improve 

the airway space in edentulous sleep apneic patients.

The qualitative analysis of the included research articles revealed that complete dentures either maintained or improved the upper airway patency [ 1 , 22 , 24 , 28 ]. Similarly, the modification of a complete denture into a MAD also improved the airway space [ 22 - 33 ]. The dentures interrupt the activation of the elevator muscles that pulls the mandible upward and forward [ 29 ]. This increases the pharyngeal patency by maintaining muscle tonicity and has a beneficial effect on the upper airway due to the decrease in obstructive events. On the contrary, it has also been stated that complete dentures occupy the tongue space, pushing it backward and thereby, narrowing the upper airway to worsen the apneic status [ 30 ]. The effectiveness of MAD was because of the anterior positioning of the tongue, and the increased lateral volume of the velopharynx due to stretching of the muscles between the soft palate and lateral pharyngeal walls [ 22 ].

We observed that the use of complete dentures did not significantly improve the quality of sleep in the reviewed articles [ 1 , 31 ]. However, contradictory results were also observed when a complete denture was used as an intervention. Chen *et al*. [ 30 ] had only 23% of individuals with daytime dysfunction while Almeida *et al*. [ 29 ] had more than 50% with daytime dysfunction due to sleep disturbance among the denture wearers. The wear of complete dentures did not improve the quality of sleep in majority of the study population although there was an improvement in upper airway space [ 1 , 29 , 31 ]. Similarly, the qualitative analysis of the reviewed articles did not reveal the influence of MAD on the quality of sleep. The reason could be attributed to the lack of adequate retention/stability of the prosthesis that caused difficulty in compliance, thus affecting sleep. Moreover, the inflammation of the soft palate and uvula was demonstrated in patients with OSA, and the presence of inflammation might be amplified due to the mucosal irritation induced by the wear of dentures/MAD in an edentulous individual [ 24 ].

Reduction in the upper airway space or the quality of sleep is attributed to the development of apneic episodes in an OSA patient. Hence, a quantitative and a qualitative analysis were done to evaluate the impact of complete dentures on the AHI index. The AHI before and after rehabilitation in the completely edentulous sleep apneic patients were assessed using PSG to confirm the type and severity of OSA [ 6 , 15 ]. A significant increase in the AHI index was observed in a few of the included studies suggesting the removal of dentures before sleep [ 29 - 31 ]. A significant reduction in the AHI index (<15) of edentulous sleep apneic patients rehabilitated with complete dentures was also observed in a few studies [ 24 , 28 ]. It was also observed that the complete dentures modified into a MAD led to the reduction in AHI in a 9-month follow-up [ 22 ]. The meta-analysis revealed that the complete denture insertion had reduced the AHI index, especially in individuals with a moderate apneic score. The outcome of the analysis was not statistically significant to consider complete denture as a definitive oral appliance to reduce sleep apnea. Cephalometric analysis in the supine position of apneic patients reported a decrease in vertical dimension due to the collapse of upper airway structures with a reduction in the retropharyngeal and posterior airway spaces [ 2 ]. Although an increase in the retropharyngeal and posterior airway spaces has been speculated after rehabilitation with complete dentures or mandibular advancement devices, the cephalometric tool could analyze only the anteroposterior diameter of the airway. Thus, a significant, but weak correlation has been found between AHI and lateral cephalometric measurements reflecting upper airway dimensions [ 2 ]. Furthermore, the complete dentures are reconstructed with an ideal maxilla-mandibular relationship, whereas the mandibular advancement device aims at guiding the mandible to position in protrusion to prevent an upper airway collapse. Hence, the lack of homogenous research studies with complete dentures modified to a MAD could have influenced the outcome of the oral appliance in elderly edentulous sleep apneic individuals in the systematic analysis.

A non-significant difference was found to exist between the complete denture wear and reduction in AHI with the diamond tracing towards the treatment effect. We speculate that the improvement of upper airway obstruction by using complete dentures to restore the vertical dimension is not as effective as expected especially in individuals suffering from a moderate or high AHI score. This systematic review suggests that the requirement of a long-term randomized controlled trial with the complete denture and MAD as an intervention in edentulous sleep apneic patients will provide in-depth detail on the effectiveness of the prosthesis. In addition, comprehensive research that analyzes AHI, upper airway space, and quality of sleep is required.

## Conclusion

Complete dentures reduce the AHI in edentulous patients with sleep apnea; however, they cannot be considered as a definitive treatment for an edentulous OSA individual. There is also inadequate evidence regarding their effectiveness in improving the airway space and quality of sleep. This systematic review demonstrates a paucity of effective evidence-based therapeutic strategies for edentulous OSA patients and the need for randomized clinical trials to assess the potential of complete dentures or MAD to improve the clinical condition.

## Acknowledgements

The authors alone are responsible for the content and writing of the paper. All authors have read and approved the final version of the manuscript. This research did not receive any specific grant from funding agency in the public, commercial or not‐for‐profit sectors.

## Conflict of Interest

The authors declare that they have no conflict of interest.
